# Effects of sex and season (breeding and non-breeding) on microhabitat selection in Stejneger’s bamboo pitviper (*Viridovipera stejnegeri*)

**DOI:** 10.7717/peerj.18970

**Published:** 2025-02-25

**Authors:** Songwen Tan, Yayong Wu, Jiajun Wang, Bing Lyu, Min Yu, He Zhang, Peng Guo, Lei Shi

**Affiliations:** 1 Faculty of Agriculture, Forestry and Food Engineering, Yibin University, Yibin, Sichuan, China; 2 Xinjiang Key Laboratory for Ecological Adaptation and Evolution of Extreme Environment Biology, College of Life Sciences, Xinjiang Agricultural University, Ulumuqi, Xinjiang, China; 3 Guizhou Academy of Forestry Science, Guiyang, Guizhou, China

**Keywords:** Microhabitat selection, Ecology, Snakes, Pitviper

## Abstract

Habitat quality and availability are crucial for the survival and reproduction of animal species. Intraspecific and seasonal differences in habitat selection reflect adaptations to changing biological requirements and environmental factors. To investigate the effects of season (breeding and non-breeding) and sex on microhabitat selection in snakes, here we employed field surveys to analyze microhabitat selection data for Stejneger’s bamboo pitviper (*Viridovipera stejnegeri*) across different sexes and seasons. Results indicated that although no significant difference was observed between groups, marked differences in certain microhabitat factors were noted. Specifically: (1) Non-breeding season females (NBF) displayed distinct differences in altitude, slope position, distance from roads compared to other groups. (2) Temperature exerted a lesser effect on non-breeding season individuals compared to breeding season individuals. Additionally, distance from roads only significantly impacted breeding season males, not females. (3) Regarding sexual differences, males and females differed in slope position and distance from residential sites, reflecting their distinct ecological requirements. Regarding seasons, differences in habitat selection between breeding and non-breeding seasons were primarily related to temperature, indicating behavioral changes linked to seasons. (4) Non-breeding season females exhibited the narrowest microhabitat niche width and the least microhabitat niche overlap with other groups, potentially due to their pronounced foraging requirements, which compel them to explore limited habitats with higher human disturbance but richer food sources. This study contributes novel insights into the habitat selection behaviors of snakes.

## Introduction

Habitat selection is the process through which animals actively choose geographical spaces that best meet their needs for predator avoidance, reproduction, and survival. Animal habitat selection is not only influenced by evolutionary and behavioral factors but also by changes in habitat quality and availability, crucial determinants of animal population survival (Ortega & Pérez-Mellado, 2016; Zhang, 2020; Zhang et al., 2023). Studying the variations in habitat selection and its influencing factors contributes to our understanding of the life histories and behavioral patterns of species (Shenbrot, 2014).

Intraspecific differences in habitat selection are common (Zhao et al., 2023), often arising due to the diverse requirements of different wild populations (Ficetola, Pennati & Manenti, 2013). For example, adult and juvenile European cave salamanders (*Hydromantes strinatii*) exhibit varied habitat preferences due to differences in risk-taking strategies (foraging and predator avoidance trade-offs; Ficetola, Pennati & Manenti, 2013). Another potential driver is intraspecific competition for resources such as food, water, and shelter, as observed in the sand lizard (*Lacerta agilis bosnica*; Popova et al., 2020) and Chinese giant salamander (*Andrias davidianus*; Zhao et al., 2023). Seasonal variations in habitat selection are also evident across multiple animal taxa, with animals adapting their habitat use in response to periodic changes in environmental factors (Rozen-Rechels et al., 2019). Therefore, habitat selection patterns fluctuate with seasonal shifts in habitat quality and availability, as well as with changes in biological behaviors and requirements (Michel et al., 2018; Bista et al., 2022), as evidenced by various species, including the lesser prairie-chicken (*Tympanuchus pallidicinctus*; Lawrence et al., 2022).

Seasonal (seasons of the year) and sexual differences in habitat selection have been confirmed in certain species of snakes. For example, the bullsnake (*Pituophis catenifer sayi*) selects different burrow habitats in response to seasonal changes in temperature and humidity (Johnson, 2021). Similarly, the canebrake rattlesnake (*Crotalus horridus*) exhibits season-based changes in habitat selection, linked to foraging, reproduction, and hibernation activities (Waldron, Lanham & Bennett, 2006). Studies on seasonal (breeding and non-breeding) differences in microhabitat selection are relatively scarce. At present, however, nocturnal arboreal snakes have been less studied due to their elusive nature and challenging habitats, which complicates data collection (Fraga et al., 2013). As such, further research is required to verify seasonal (breeding and non-breeding) and sexual differences in habitat selection among nocturnal arboreal snake species.

Existing methods for surveying snake habitat selection typically include: fixed-distance transect or quadrat methods (Mazerolle et al., 2007), comparisons between presence points and pseudo-absence points (Johnson et al., 2006), and radio tracking is used to obtain presence data, which is then compared to unrecorded pseudo-absence data (Whitaker & Shine, 2003). Furthermore, recent studies have frequently employed repeated sampling of the same transects and individuals, as well as habitat sampling at multiple locations near individuals (Zhang et al., 2020). However, due to the cryptic nature of snakes, detecting individuals is often the most challenging issue. Additionally, the limited density of snakes results in insufficient data for statistical analysis, making repeated sampling one of the primary reasons for data deviation. Subjectivity in sampling is also a significant factor contributing to data discrepancies. Various analytical methods for habitat selection have emerged, such as calculating habitat selection using Vanderploeg (*W*_*i*_) and Scavia (*E*_*i*_) selection indices, or predicting habitat use through logistic regression, resource selection function, random forest model, and generalized linear mixed model (Zhang et al., 2020). Furthermore, in order to mitigate the influence of volatile factors such as temperature and humidity, a single visit occupancy model has been developed and applied (Sólymos & Lele, 2016).

The nocturnal arboreal Stejneger’s bamboo pitviper (*Viridovipera stejnegeri*, Squamata, Viperidae) is widely distributed in southern China and Vietnam (Guo et al., 2022). This species primarily resides in rocky cliffs and vegetated areas near mountain streams, creeks, and other wet environments, subsisting mainly on frogs, but also consuming lizards and small rodents (Creer et al., 2002). The reproductive mode of this species is ovoviviparity, typically giving birth to 5–8 neonates in August, with the offspring measuring approximately 250 mm in total length (Zhao, 2006). Previous research has indicated that the species showed no significant differences in vegetation-based habitat selection between males and females (Tu, Wang & Lin, 2000). Therefore, we hypothesize that there are no significant seasonal or sexual differences in microhabitat selection of *V. stejnegeri*. This study aims to investigate whether there are differences in microhabitat selection of *V. stejnegeri* among different seasons and genders.

## Materials and Methods

### Study area

**Figure 1:**
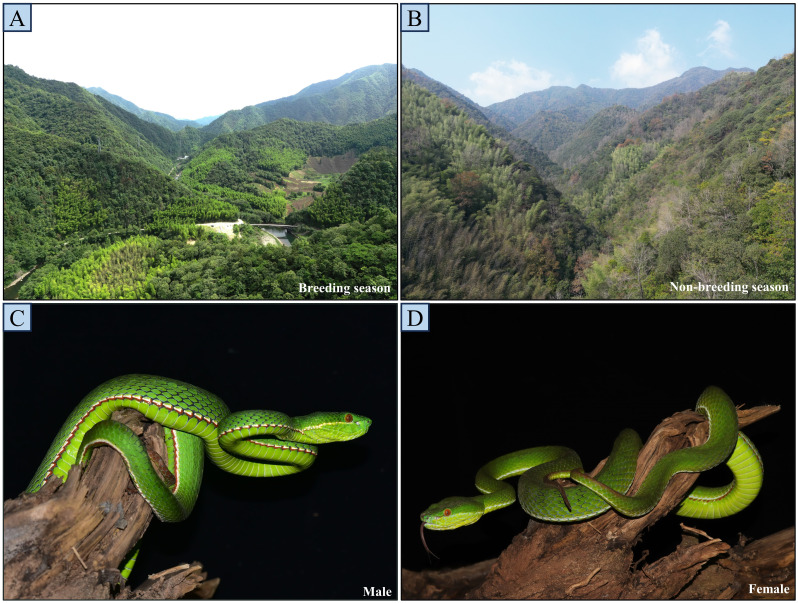
Breeding and non-breeding season habitats of the study site (A and B), and general view of a male (C) and female (D). The photos A and B were taken from adjacent locations within the same area (200 m apart).

Data were collected in Huangshan City, Anhui Province, eastern China (117°23′–118°55′E, 29°24′–30°24′N; Fig. 1), the study area covers 25 square kilometers. The region is characterized by a subtropical monsoon climate with abundant heat and moisture. Annual average temperature ranges from 15.5 to 16.4 °C, and average annual precipitation ranges from 1,395 to 1,702 mm, predominantly falling from May to August (summer), with reduced rainfall from September to November (autumn), although it does not reach an extreme level of aridity (The People’s Government of Anhui Province, 2023). The vegetation is primarily composed of subtropical evergreen broad-leaved forests, the understory is primarily composed of perennial herbs (Poaceae and Urticaceae) and ferns, with minimal variation in vegetation diversity between seasons.

### Data acquisition and variable assignment of categories

Data were collected during two periods, June–July 2023 and September–October 2023, from approximately 20:00 to 24:00. We extensively searched for *V. stejnegeri* in the survey area, and manually collected snakes. Each located snake was injected with an independent electronic tag (EM4305; wnLikes, Shenzhen, China), and its unique identification number, latitude and longitude coordinates, and sex were recorded. Previous studies on the closely related species *Trimeresurus macrops*, have revealed that this group typically exhibits a relatively small hunting range and home range. Therefore, reference to *T. macrops*, at each sampling location, a 4 × 4 m quadrat was established (Strine et al., 2018). Within these quadrats, 13 habitat factors were measured, with detailed descriptions and measurement methods provided in Appendix S1. A total of 92 snakes were collected (females = 34, males = 58), all snakes were released after data collection. The data were obtained only at the time of the first collection.

Sex identification was performed *via* cloacal probing by gently pressing the region located five cm below the vent at the tail towards the anterior direction of the cloaca using the thumb. The detection of an everted hemipenis was used to determine the sex of the individual. Existing research indicated that *V. stejnegeri* typically given birth in August (Huang, 1980; Huang et al., 2015). Therefore, data collected between June and July were designated as breeding season data, while data from September to October were considered non-breeding season data. During each survey period, ArcGIS v10.8 was used to randomly generate 50 pseudo-absence points within the survey area, excluding unsuitable habitats for *V. stejnegeri* such as highways, open water bodies, and cliffs, as well as points with the presence of *V. stejnegeri* within a 50-meter radius (Mizsei et al., 2024). The remaining points served as pseudo-absence points for measuring the aforementioned habitat factors (Zhang, 2020). All animal procedures were carried out in accordance with and approved by the Animal Care and Use Committee at Yibin University (animal protocol number: YBU2020007). We also adhered to the ARROW (Animals in Research: Reporting On Wildlife) guidelines.

### Data processing and analysis

The collected data were categorized into four groups based on season and sex: breeding season males (BM), breeding season females (BF), non-breeding season males (NBM), and non-breeding season females (NBF).

Before starting data analysis, we checked and removed all extreme outliers and used the chain equation multivariate interpolation method to impute missing values. And all data were logarithmically processed before analysis to improve normality. All data were tested for normality and homogeneity of variance.

To analyze the habitat selection preferences of *V. stejnegeri* for each habitat factor, the Vanderploeg (*W*_*i*_) and Scavia *(E*_*i*_) selection indices were calculated (Vanderploeg & Scavia, 1979):

\begin{eqnarray*}{W}_{i}& =( \frac{{r}_{i}}{{p}_{i}} )/\sum \left( \frac{{r}_{i}}{{p}_{i}} \right) \end{eqnarray*}

\begin{eqnarray*}{E}_{i}& =({W}_{i}- \frac{1}{n} )/({W}_{i}+ \frac{1}{n} ) \end{eqnarray*}

where *r*_*i*_ denotes the number of quadrats selected by *V. stejnegeri* with characteristics *i*; *p*_*i*_ denotes the total number of quadrats in the environment with characteristics *i*; and *n* refers to the level or category number of a particular habitat factor. *E*_*i*_ ranges from −1 to 1, with *E*_*i*_ = 1 indicating strong preference, 0.1 < *E*_*i*_ < 1 indicating selection, −0.1 < *E*_*i*_ < 0.1 indicating random selection, −1 < *E*_*i*_ < −0.1 indicating avoidance, and *E*_*i*_ = −1 indicating no selection.

To analyze the importance of habitat factors for each group, factor autocorrelation analysis was performed to exclude highly correlated factors (see Appendix S3). Random forest models were then constructed using the habitat data for each group as well as the pseudo-absence points data. We used viper presence/pseudo-absence as the dependent variable and employed the R package “randomForest” to construct random forest models, employed the mean decrease Gini index to evaluate the importance of each factor (Zhang et al., 2020).

Multiple linear models were used to analyze the interactive effects of season and sex on various factors. For factors showing significant effects, *post hoc* multiple comparisons (Least significant difference, LSD) were conducted among the four groups, while for factors without significant effects, differential analyses were restricted to within either seasonal or sexual groups. Prior to within-group analyses, tests for normality and homogeneity of variance were conducted on continuous variables. Continuous variables that met the assumptions of normality and homogeneity of variance were analyzed using one-way analysis of variance (ANOVA), while those that did not meet these criteria were analyzed using the Mann–Whitney U test. Discrete variables were examined using chi-square tests to identify differences within groups. We also calculated the mean and standard deviation within each group. Furthermore, partial dependence plots were employed to visually depict the relationship between habitat factors and predicted probability of occurrence for each group, as determined from the random forest models (Hastie, Tibshirani & Friedman, 2005).

To analyze overall differences and associations in microhabitat selection among groups, the microhabitat niche width Levins index (*B*) for each group was calculated using the *spaa* package in R (R Development Core Team, 2018):

\begin{eqnarray*}B= \frac{1}{\sum _{j=1}^{R}{ \left( {P}_{j} \right) }^{2}} \end{eqnarray*}

where *P*_*j*_ represents the proportion of individuals found at sampling point *j* and *R* denotes the total number of sampling points (Simpson, 1949). Subsequently, microhabitat niche overlap between groups was quantified using the Levins index (*O*_*ik*_):

\begin{eqnarray*}{O}_{ik}= \frac{\sum _{j=1}^{R}{P}_{ij}{P}_{kj}}{\sum _{j=1}^{R}{ \left( {P}_{ij} \right) }^{2}} \end{eqnarray*}

where *P*_*ij*_ and *P*_*kj*_ represent the abundance of groups *i* and *k* at sampling point *j*, respectively, and *R* is the total number of sampling points (Levins, 1968).

To calculate the significance of niche overlap, we randomly resampled data regardless of group identity sort them to two group, calculate niche overlap and compare observed value by a *t*-test.

Principal component analysis (PCA) was conducted to determine the eigenvalues of each principal component (PC). PCA scatter plots were created using the PCs with the highest eigenvalues as axes. The distribution of points within 95% confidence ellipses for different groups on the scatter plot was analyzed to assess whether significant differences existed among the groups. To further validate the results, permutational multivariate analysis of variance (PERMANOVA) (ADONIS) was conducted using the *vegan* package in R, with the degree of overlap among groups in the graphical representations assessed to determine whether significant differences existed (R Development Core Team, 2018).

All analyses were conducted with a confidence level of 0.05. Data analysis was performed using R v4.2.3, and graphs were plotted using R v4.2.3 and Origin 2022 (R Development Core Team, 2018).

## Results

### Microhabitat preferences and factor importance among groups

A total of 62 presence points were collected during the breeding season (males = 43, females = 19) and 30 during the non-breeding season (males = 15, females = 15), all snakes are adults (SVL: 57.04 ± 5.19 cm).

**Figure 2:**
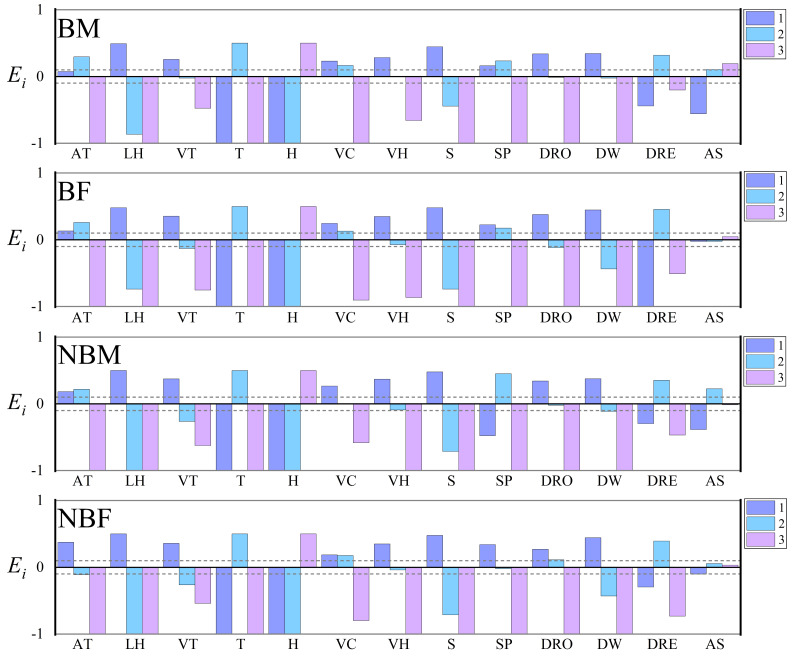
Microhabitat selection among four groups of *V. stejnegeri*. AT, altitude: 1. <200 m; 2. 200–400 m; 3. >400 m; LH, landscape habitat: 1. Stream; 2. Forest; 3. Agricultural; VT, vegetation type: 1. Grass; 2. Shrub; 3. Tree; T, temperature: 1. <20 °C; 2. 20–30 °C; 3. >30 °C; H, humidity: 1. <40%; 2. 40%–70%; 3. >70%; VC, vegetation coverage: 1. <20%; 2. 20%–70%; 3. >70%; VH, vegetation height: 1. 0–2 m; 2. 2–5 m; 3. >5 m; S, slope: 1. 0°–15°; 2. 15°–40°; 3. >40°; SP, slope position: 1. Down-slope position; 2. Mid-slope position; 3. Up-slope position; DRO, distance from roads: 1. 0–10 m; 2. 10–30 m; 3. >30 m; DW, distance from water: 1. <5 m; 2. 5–20 m; 3. >20 m; DRE, distance from residential sites: 1. <100 m; 2. 100–500 m; 3. >500 m; AS, aspect: 1. Sunny slope (135° half-shaded and half-sunny slope (45°–135°, 225°–315°). *E*_*i*_ is the Scavia selection index. *E*_*i*_ = 1 indicating strong preference, 0.1 < *E*_*i*_ < 1 indicating selection, −0.1 < *E*_*i*_ < 0.1 indicating random selection, −1 < *E*_*i*_ < −0.1 indicating avoidance, and *E*_*i*_ = −1 indicating no selection. The dashed lines represent marker lines at 0.1 and −0.1.

The Vanderploeg (*W*_*i*_) and Scavia (*E*_*i*_) selection indices revealed no significant differences among the four groups in their preferences for factors such as temperature, humidity, vegetation height, vegetation coverage, slope, distance from water, distance from residential sites, landscape habitat, and vegetation type. However, compared to other the groups, NBF displayed a preference for lower altitudes and slopes, and for locations furthest from roads. Additionally, all groups showed either no preference or extremely weak preference for “aspect” factor, leading to its exclusion in subsequent analyses (Fig. 2).

**Figure 3:**
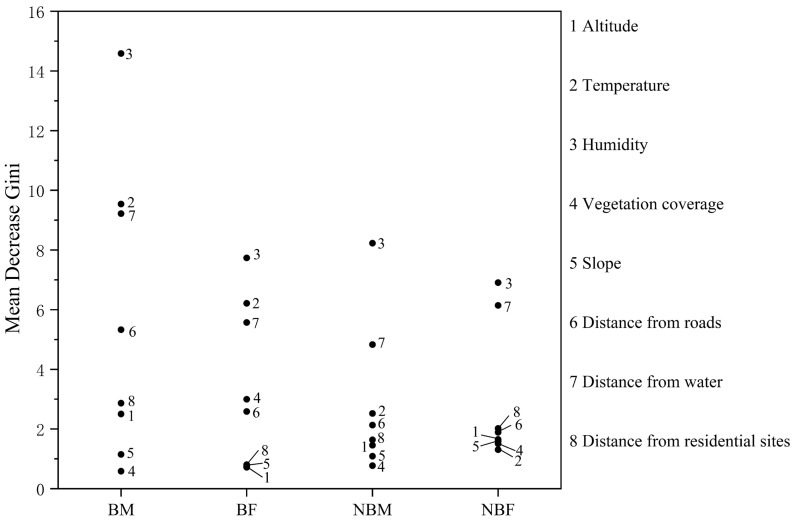
Factor importance in microhabitat selection by *V. stejnegeri*.

The mean decrease Gini index identified the key factors influencing habitat selection. For the BM group, important factors included humidity, temperature, distance from water, and distance from roads. The BF group was influenced by humidity, temperature, and distance from water, while the NBM and NBF groups were influenced by humidity and distance from water (Fig. 3).

### Seasonal (breeding and non-breeding) and sexual differences

Multiple linear model analyses revealed that, apart from altitude, there were no significant interactions between sex and season for other factors (Appendix S2).

**Figure 4:**
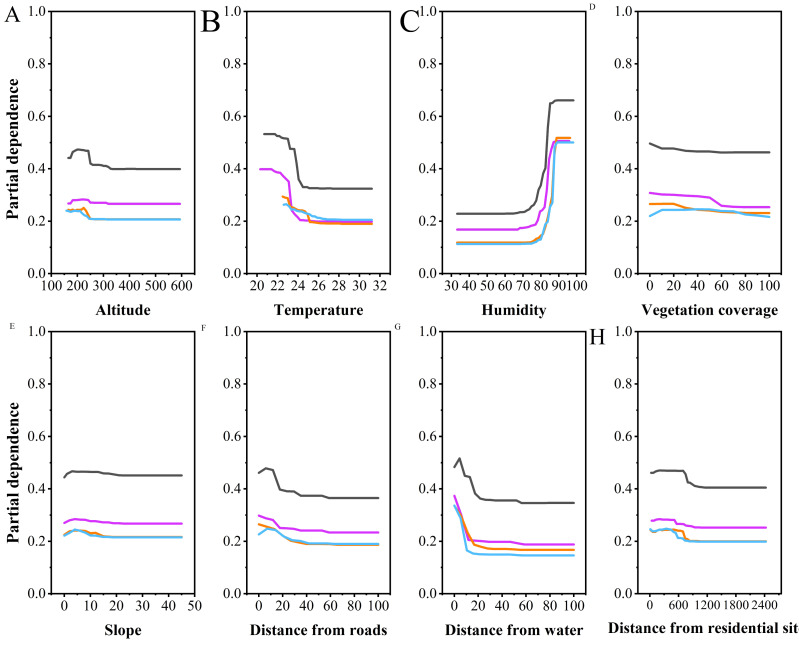
Partial dependence plots of various habitat factors based on random forest model. Y-axes are partial dependence (dependence of the probability of occurrence on one predictor variable after averaging out effects of the other predictor variables in the model). Black represents breeding season males, purple represents breeding season females, orange represents non-breeding season males, and blue represents non-breeding season females.

Mann–Whitney U and chi-square tests demonstrated significant differences between males and females only in distance from residential sites (M *vs* F: 414.98 ± 170.96 *vs* 338.65 ± 148.48; *P* = 0.026) and slope position (F *vs* M: mid-slope position *vs* down-slope position; *P* = 0.004; Table 1). Seasonal differences analyses only indicated a significant difference in temperature preference (M *vs* F: 21.95 ± 0.81 *vs* 23.71 ± 0.84; *P* < 0.001; Table 1), with no significant differences in preferences for other factors. Specific differences among groups are illustrated in Fig. 4.

**Table 1:** Seasonal and sexual differences in microhabitat selection.

**Variable**	**Sex (M** **= 58, F = 3 4)**				**Season (B = 62, NB = 30)**			
	**Mann–Whitney U test**		**Chi-square test**		**Mann–Whitney U test**		**Chi-square test**	
	***P***	***Z***		*P*		*χ2*		***P***	***Z***		*P*	*χ2*
Temperature	0.094		−1.676				<0.001	−6.726		
Humidity	0.610		0.510				0.088	−1.704		
Vegetation height	0.864		0.171				0.725	0.352			
Vegetation coverage	0.906		0.119				0.065	−1.845		
Slope	0.184		1.329				0.465	0.730			
Distance from roads	0.164		−1.390				0.079	−1.754		
Distance from water	0.053		1.932				0.134	−1.499		
Distance from residential sites	0.026		2.233				0.917	0.104			
Landscape habitat			0.672		1.055			0.549	1.453
Vegetation type			0.943		0.117			0.603	1.013
Slope position			0.004		8.289			0.747	0.104

**Notes.**

M males F females B breeding season NB non-breeding season

Multiple comparisons analysis revealed that the altitudes selected by NBF differed significantly from those selected by NBM (*P* < 0.001), BM (*P* = 0.001), and BF (*P* = 0.009). However, there were no significant differences in altitude selection among NBM, BM, and BF. Partial dependence plots indicated that NBF selected lower altitudes than the other groups, with a narrower range of altitude preferences (NBF: 190.73 ± 19.06, BM: 212.09 ± 15.91, NBM: 211.42 ± 17.63, BF: 208.40 ± 22.77, Fig. 4A).

### Overall differences in microhabitat selection among different groups

Microhabitat niche analysis indicated that the microhabitat niche widths varied among the different groups, with NBM exhibiting the largest (2.935) and NBF the smallest (1.895). As shown in Table 2, the greatest microhabitat niche overlap was found between NBM and BF (98.63%), while the smallest overlap was found between NBF and BM (53.86%). The results of the paired sample *t*-test between randomly resampling and observed values show that the niche overlap index of the observed values is slightly lower than that of randomly resampling (79.74 ± 18.60 *vs* 85.61 ± 14.89), but the difference is not significant (*t* = 0.533, *P* = 0.617). This indicates that niche differentiation among the groups is not pronounced.

**Figure 5:**
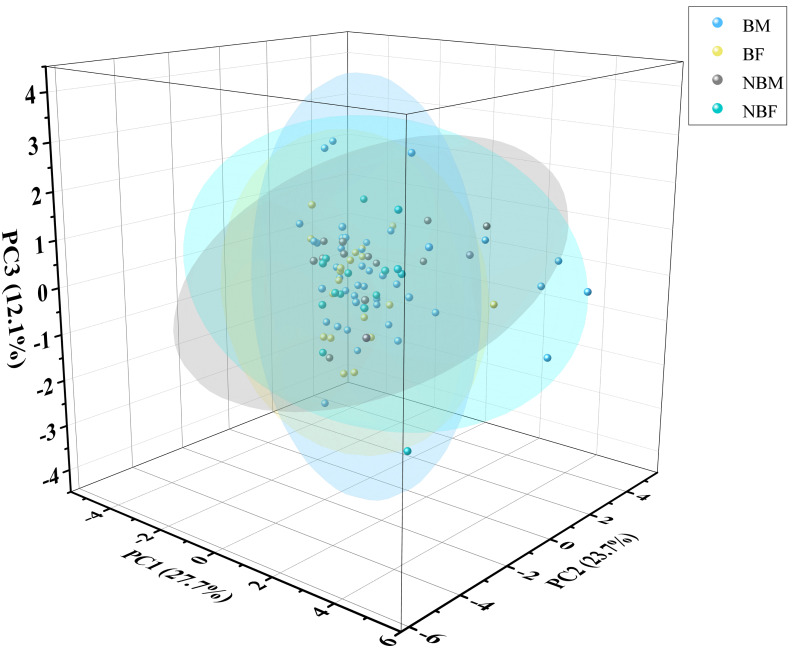
PCA of microhabitat selection by four groups of *V. stejnegeri*.

**Figure 6:**
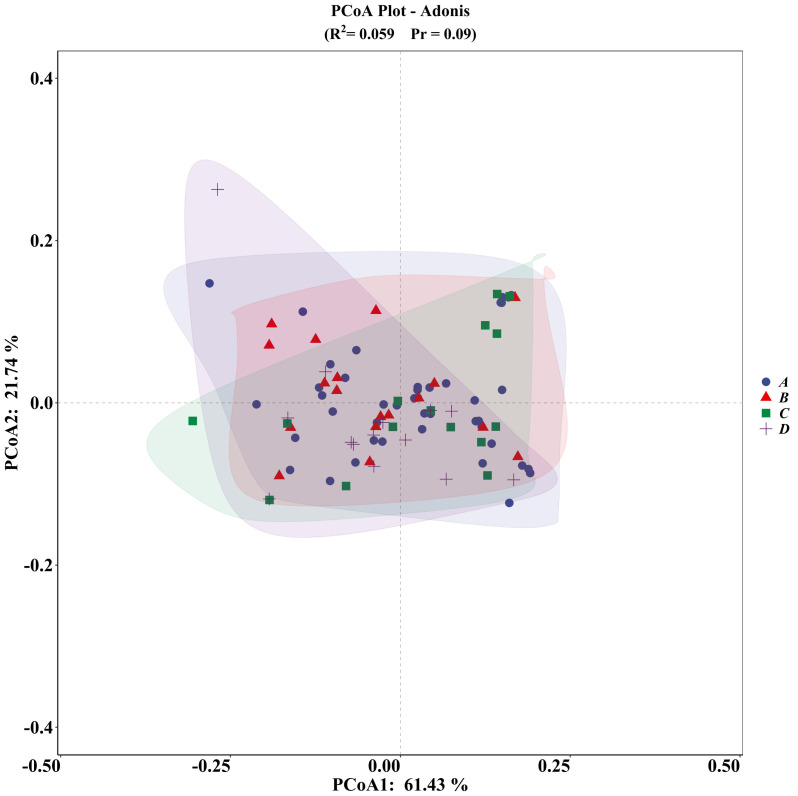
ADONIS analysis of microhabitat selection by four groups of *V. stejnegeri*. (A) represents breeding season males, (B) represents breeding season females, C represents non-breeding season males, and D represents non-breeding season females.

The PCA plot indicated no significant differences among the four groups (Fig. 5), supported by ADONIS analysis (Fig. 6).

## Discussion

Sexual and seasonal (breeding and non-breeding) differences in habitat selection have been extensively documented across various animal taxa (Ficetola, Pennati & Manenti, 2013; Popova et al., 2020; Zhao et al., 2023). Sexual differences are usually driven by varying needs between the sexes or intense intersexual competition. Seasonal differences typically relate to changes in the availability or quality of resources, as well as shifts in animal behavior and requirements throughout the year. In this study, sexual and seasonal differences in microhabitat selection in *V. stejnegeri* were explored. The findings showed that although there were no pronounced overall differences among the groups, there were specific differences in preferences for certain factors, thereby not supporting our hypothesis and contradicting previous research (Tu, Wang & Lin, 2000). While the earlier study by Tu, Wang & Lin (2000) examined sexual differences in vegetation utilization characteristics, it did not explore other factors or seasonal differences. As such, our research provides a more comprehensive analysis, contributing to a deeper understanding of the life activities of this species.

**Table 2:** Microhabitat niche overlap among groups of *V. stejnegeri*.

		BF	BM	NBF	NBM
Observed value	BF	/	97.51	84.75	98.63
	BM	97.51	/	53.86	82.74
	NBF	84.75	53.86	/	60.96
	NBM	98.63	82.74	60.96	/
Randomly resample	BF	/	82.08	65.17	83.72
	BM	82.08	/	78.20	96.48
	NBF	65.17	78.20	/	108.00
	NBM	83.72	96.48	108.00	/

Vanderploeg (*W*_*i*_) and Scavia (*E*_*i*_) selection indices revealed differences in preferences for altitude, slope position, and distance from roads between NBF and the other groups, possibly due to the stronger foraging needs of these individuals. Notably, females often experience significant energy depletion after reproductive activities, prompting them to select habitats with higher predation success rates (Harvey & Weatherhead, 2010). In the study area, the non-breeding season coincides with the dry season, during which potential food sources for *V. stejnegeri*, such as frogs, tend to congregate in downstream areas with sufficient water and significant human (agricultural) disturbances. Therefore, NBF displayed a preference for downhill positions and lower altitudes, possibly taking risks to meet higher foraging requirements. This behavior is consistent with the results obtained from multiple comparison analyses and random forest model predictions (Fig. 4). Similar risk-taking strategies driven by foraging demands have also been observed in other species, such as *Hydromantes strinatii* (Ficetola, Pennati & Manenti, 2013) and *Trimeresurus macrops* (Strine et al., 2018), although these studies did not differentiate between various groups within the species. The differentiation among groups in our study provides a basis for further discussions on the varied impacts of human disturbances on different snake groups.

Regarding factor importance, the influence of temperature on microhabitat selection for non-breeding season individuals was relatively minor compared to that of breeding season individuals (Fig. 3). This may stem from slightly higher nighttime average temperatures during the non-breeding season compared to the breeding season in the study area (23.71 ± 0.84 *vs* 21.95 ± 0.81). Therefore, it easier for snakes to find habitats with suitable temperatures during the non-breeding season. Nocturnal snakes typically require less precise thermoregulation and often adopt a passive thermoregulation strategy (Vitt, Zani & Lima, 1997). In the non-breeding season, higher environmental temperatures facilitate thermoregulation in *V. stejnegeri*, thereby reducing the impact of temperature on microhabitat selection compared to the breeding season. Additionally, proximity to roads, typically associated with shelter due to roadside crevices serving as ideal hides for snakes (Shew, Greene & Durbian, 2012), showed an unexpected pattern in our study. Notably, shelters proved more crucial resource for males than females during the breeding season, contrasting with trends observed in northern pine snakes (*Pituophis m. melanoleucus*; Gerald, Bailey & Holmes, 2006) and northern Mexican gartersnakes (*Thamnophis eques megalops*; Sprague & Bateman, 2018). One possible explanation is that, similar to eastern brown snakes (*Pseudonaja textilis*; Whitaker & Shine, 2003), males exhibit strong territorial behavior towards shelters during the breeding season. Alternatively, NBF may select trees away from roads as shelters to minimize human disturbance, which is consistent with the findings of Strine et al. (2018). Additionally, roads typically have higher nighttime temperatures, so the distance from roads may also be related to the competition among males for thermal resources.

Minimal seasonal and sexual variation in microhabitat selection was detected in *V. stejnegeri*, with only a few habitat factors showing differences. Significant sexual differences were only found in slope position and distance from residential sites, likely due to different reproductive needs of males and females. Males expanding their habitat range can increase encounter rates with females and reduce intrasexual reproductive competition, thereby enhancing mating opportunities (Shew, Greene & Durbian, 2012). In contrast, females require stable and suitable habitats for reproductive activities, as reported in other species such as Chinese giant salamanders (Zhao et al., 2023). Significant differences in temperature preferences between the breeding season and non-breeding season were also detected for *V. stejnegeri*, while other factors remained consistent, aligning with the findings from factor importance analysis.

In terms of microhabitat niche analysis, NBF exhibited the narrowest microhabitat niche width and lowest microhabitat niche overlap compared to the other groups. This pattern is potentially due to the strong foraging demands of these females, leading them to forage in limited habitats with higher human disturbance but richer food resources. Overall, the PCA and ADONIS results showed no significant differences among the groups. However, a notable limitation is that all females surveyed were either non-pregnant or in early pregnancy (undeveloped eggs; Survey: June, Birthing: August), preventing a comparison of microhabitat selection preferences between pregnant and non-pregnant females. Although this limitation has been acknowledged in previous research (Shew, Greene & Durbian, 2012), many studies have documented significant differences in habitat selection between pregnant and non-pregnant females (*e.g.*, Blouin-demers & Weatherhead, 2001; Harvey & Weatherhead, 2006; Crane & Greene, 2008). Some of our factors, such as temperature and humidity, exhibit characteristics of continuous fluctuation; therefore, a single measurement may exert a certain influence on the results. In the future, we will employ new analytical methods to optimize our outcomes, such as the single visit occupancy model (Sólymos & Lele, 2016). Furthermore, despite our efforts to collect relevant microhabitat data, many factors influencing microhabitat selection, such as the significant influence of canopy height observed in *T. macrops* (Barnes, Strine & Suwanwaree, 2023), were not collected. These unexplored factors could lead to noticeable differences among groups, potentially resulting in ecological niche divergence. Therefore, more comprehensive data collection is required in our future research.

## Conclusions

This study investigated sexual and seasonal (breeding and non-breeding) differences in microhabitat selection of *V. stejnegeri*. While no significant overall differences were observed among groups, certain microhabitat factors did show variations. Specifically, NBF differed from the other groups in terms of preference for altitude, slope position, and distance from roads. Regarding factor importance, temperature had a lesser effect on non-breeding season individuals compared to breeding season individuals. Furthermore, distance from roads significantly influenced BM but not BF. Regarding sexual differences, males and females varied in preferences for slope position and distance from residential sites, reflecting differing requirements between the sexes. Seasonally, the primary differences in habitat selection between the breeding and non-breeding seasons were in temperature, influenced by seasonal behavioral changes. Regarding microhabitat niches, NBF exhibited the narrowest microhabitat niche width and lowest microhabitat niche overlap with the other groups. We speculate that this is due to the strong foraging requirements of these females, leading them to venture into limited habitats with higher human disturbances but richer food sources. Overall, this study offers new insights into the habitat selection of snakes, thus enhancing our understanding of their ecological preferences.

## Supplemental Information

10.7717/peerj.18970/supp-1 Supplemental Information 1 Code The file can be opened using any version of R or RStudio. Some of the analysis was conducted using SPSS 27.

10.7717/peerj.18970/supp-2 Supplemental Information 2 Raw data

10.7717/peerj.18970/supp-3 Supplemental Information 3 ARRIVE 2.0 Checklist

10.7717/peerj.18970/supp-4 Supplemental Information 4 Appendix 1 Description and categories of habitat factors

10.7717/peerj.18970/supp-5 Supplemental Information 5 Appendix 2 Influence of sex, season, and their interactions on selection of various habitat factors

10.7717/peerj.18970/supp-6 Supplemental Information 6 Appendix 3 Autocorrelation of habitat factors for microhabitat selection by *V. stejnegeri* A, altitude; T, temperature; H, humidity; VC, vegetation coverage; VH, vegetation height; S, slope; DRO, distance from roads; DW, distance from water; DRE, distance from residential sites.

## Additional Information and Declarations

### Data Availability

The following information was supplied regarding data availability: Raw data and code are available in the Supplemental Files.
