# Supplementary material for: Effects of sex and season (breeding and non-breeding) on microhabitat selection in Stejneger’s bamboo pitviper (Viridovipera stejnegeri)
Source: PeerJ. 2025 Feb 25;13:e18970. doi: 10.7717/peerj.18970 (PMC11869892; doi:10.7717/peerj.18970)
Supplement: Supplemental Information 4 [file peerj-13-18970-s004.docx]

**Appendix 1 Description and categories of habitat factors**

| Variable | Description | Categories |
| --- | --- | --- |
| Altitude (m) | Altitude in quadrat was directly measured by GPS. | 1. < 200 m; 2. 200–400 m; 3. > 400 m. |
| Temperature (℃) | Ambient temperature in quadrat was measured using a portable hand-held electronic temperature and hygrometer (DL333006, +0.1℃; Deli, Ningbo, China). During measurement, thermohygrometer was placed on the substrate where the snake is located. Once values stabilized, readings were recorded. Procedure was repeated three times consecutively, and average value was calculated. | 1. < 20 ℃; 2. 20–30 ℃; 3. > 30 ℃. |
| Humidity (%) | Ambient humidity in quadrat was measured using a portable hand-held electronic temperature and hygrometer (DL333006, +0.1%RH; Deli, Ningbo, China). Measurement was analogous to temperature measurement. | 1. < 40%; 2. 40%–70%; 3. > 70%. |
| Landscape habitat | Habitat types within 300 m of quadrat were assessed. | 1. Stream; 2. Forest; 3. Agricultural. |
| Vegetation type | Main vegetation types in quadrat were assessed. | 1. Grass; 2. Shrub; 3. Tree. |
| Vegetation height (m) | Vegetation height of herbs and shrubs was measured by tape measure, arbor height was calculated by trigonometric function. We categorized based on the height of shrubs and herbaceous plants, broadleaf trees, and coniferous trees in the study area. | 1. 0–2 m; 2. 2–5 m; 3. > 5 m. |
| Vegetation coverage (%) | when the sun was positioned highest above the horizon, long (a) and short diameters (b) of shadows were measured with a tape measure, then calculated using the formula 1/4πab. | 1. < 20%; 2. 20%–70%; 3. > 70%. |
| Slope (°) | Average slope of quadrat was measured using a handheld GPS. | 1. 0°–15°; 2. 15°–40°; 3. > 40°. |
| Slope position | Slope position is estimated based on the location of the quadrat within the entire hillside. | 1. Down-slope position; 2. Mid-slope position; 3. Up-slope position. |
| Aspect (°) | Quadrat aspect was recorded with GPS. And classification of different aspect categories based on the duration of solar radiation throughout the day. | 1. Sunny slope (135° half-shaded and half-sunny slope (45°–135°, 225°–315°). |
| Distance from roads (m) | Distance of quadrat from the road (m) was measured using a tape measure. | 1. 0–10 m; 2. 10–30 m; 3. > 30 m. |
| Distance from water (m) | Distance of quadrat from water was measured using a tape measure. | 1. < 5 m; 2. 5–20 m; 3. > 20 m. |
| Distance from residential sites (m) | Distance of quadrat from residential sites (The nearest residential buildings on the map) was measured using the ArcGIS v10.8 Mapping tool. | 1. < 100 m; 2. 100–500 m; 3. > 500 m. |
