# Supplementary material for: Effects of sex and season (breeding and non-breeding) on microhabitat selection in Stejneger’s bamboo pitviper (Viridovipera stejnegeri)
Source: PeerJ. 2025 Feb 25;13:e18970. doi: 10.7717/peerj.18970 (PMC11869892; doi:10.7717/peerj.18970)
Supplement: Supplemental Information 5 [file peerj-13-18970-s005.docx]

**Appendix 2 Influence of sex (males and females), season (breeding and non-breeding), and their interactions on selection of various habitat factors**

| **Variable** | **Sex** | | **Season** | | **Sex × Season** | |
| --- | --- | --- | --- | --- | --- | --- |
|  | ***F*** | ***P*** | ***F*** | ***P*** | ***F*** | ***P*** |
| Altitude | 4.950 | 0.029 | 8.750 | 0.004 | 4.251 | 0.042 |
| Landscape habitat | 0.233 | 0.631 | 0.233 | 0.631 | 0.233 | 0.631 |
| Temperature | 0.754 | 0.388 | 86.942 | 0.001 | 0.571 | 0.452 |
| Humidity | 0.008 | 0.928 | 5.075 | 0.027 | 0.316 | 0.576 |
| Vegetation type | 0.015 | 0.903 | 0.332 | 0.566 | 0.738 | 0.393 |
| Vegetation height | 0.021 | 0.884 | 0.016 | 0.900 | 1.488 | 0.226 |
| Vegetation coverage | 0.483 | 0.489 | 3.338 | 0.071 | 1.484 | 0.226 |
| Slope | 1.145 | 0.288 | 0.405 | 0.526 | 0.004 | 0.951 |
| Slope position | 5.572 | 0.020 | 0.416 | 0.521 | 1.430 | 0.235 |
| Distance from roads | 1.170 | 0.282 | 5.103 | 0.026 | 1.121 | 0.293 |
| Distance from water | 4.037 | 0.048 | 1.776 | 0.186 | 0.091 | 0.764 |
| Distance from residential sites | 3.918 | 0.051 | 0.001 | 0.982 | 0.026 | 0.871 |
