# Supplementary figures and images for: Effects of sex and season (breeding and non-breeding) on microhabitat selection in Stejneger’s bamboo pitviper (Viridovipera stejnegeri)
Source: PeerJ. 2025 Feb 25;13:e18970. doi: 10.7717/peerj.18970 (PMC11869892; doi:10.7717/peerj.18970)

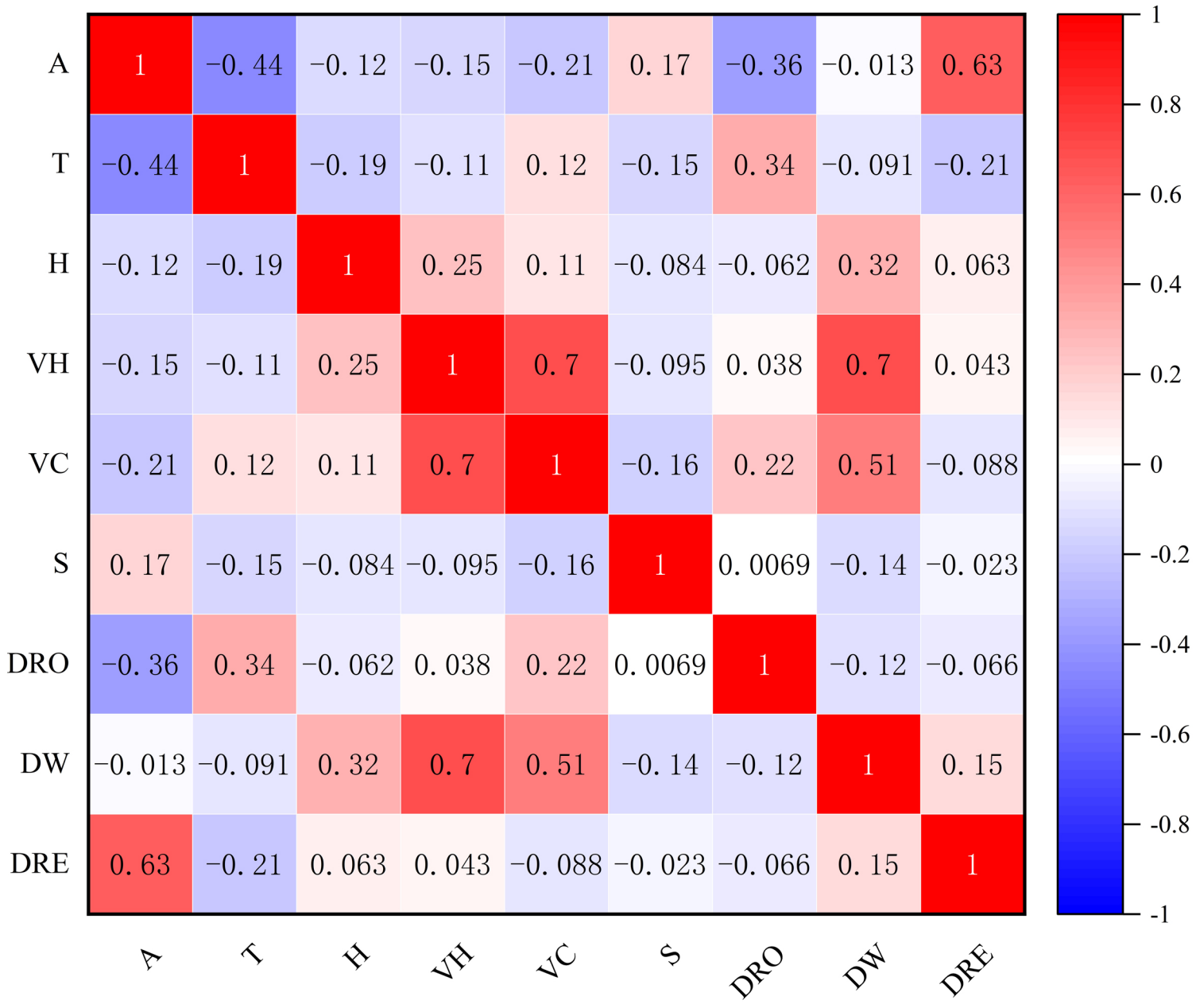

Supplement: Supplemental Information 6 — A, altitude; T, temperature; H, humidity; VC, vegetation coverage; VH, vegetation height; S, slope; DRO, distance from roads; DW, distance from water; DRE, distance from residential sites. [file peerj-13-18970-s006.pdf]
